# PETRA: Drug Engineering via Rigidity Analysis

**DOI:** 10.3390/molecules25061304

**Published:** 2020-03-12

**Authors:** Sam Herr, Josh Myers-Dean, Hunter Read, Filip Jagodzinski

**Affiliations:** Western Washington University, Computer Science, Bellingham, WA 98225, USA

**Keywords:** ligand engineering, rigidity analysis

## Abstract

Rational drug design aims to develop pharmaceutical agents that impart maximal therapeutic benefits via their interaction with their intended biological targets. In the past several decades, advances in computational tools that inform wet-lab techniques have aided the development of a wide variety of new medicines with high efficacies. Nonetheless, drug development remains a time and cost intensive process. In this work, we have developed a computational pipeline for assessing how individual atoms contribute to a ligand’s effect on the structural stability of a biological target. Our approach takes as input a protein-ligand resolved PDB structure file and systematically generates all possible ligand variants. We assess how the atomic-level edits to the ligand alter the drug’s effect via a graph theoretic rigidity analysis approach. We demonstrate, via four case studies of common drugs, the utility of our pipeline and corroborate our analyses with known biophysical properties of the medicines, as reported in the literature.

## 1. Introduction

The drug discovery and development process has advanced greatly over the last few decades. This is in part due to both an increased understanding of the biochemistry and biophysics of protein-ligand interactions and to the improvement in wet-lab methods of in vitro screening of novel drugs. Wet-lab methods offer the option to scan through thousands of compounds using techniques, such as high-throughput screening [1] or deep mutational scanning [2]. With this increased understanding of protein-ligand interactions, many novel approaches to drug design have been created both in silico and in vitro. These approaches include exploring enzyme flexibility [3], maximizing ligand affinity and selectivity [4,5], the use of templates [6], and the use of homology modeling [7]. A diverse variety of computational approaches [8], accessible either as libraries or via servers, are available. They include LEA3D [9], LigPlot+ [10], iScreen [11], e-LEA3D [12], iSmart [13], and PharmMapper [14]. These approaches employ a variety of techniques to better understand the effects of a ligand on a protein. Among them are fragment-based modeling approaches, which have been on the rise for the last decade [15]. Pharmacophore methods, which focus on the chemical activity of the ligand and the interactions between the proteins and ligand, have been helpful in aiding the drug discovery process. However, these methods need to still be improved in efficiency and efficacy in order to be used with high success in the drug design process [16]. Approaches similar to pharmacophore-based techniques include QSAR (quantitative structural-activity relationship), which reasons that structurally similar compounds show similar biological activity. Chemical and physical interactions, such as hydrophobic interactions, are used as correlation markers for biological activity [17]. Web services are also available, including e-LEA3D, which allows a user to in silico create new ligands based on a customizable fitness score. The score can be based on molecular properties or a protein-ligand docking score [12].

There still remains a high percentage of late stage failures in clinical development that is attributed to safety and efficacy concerns [18]. The safety and efficacy of a drug is partly determined in the early stages of drug development, where computational efforts can be used to select the best drug candidates with respect to certain parameters [17]. However, Lange et al. believe “early drug research efforts are too reductionist” in terms of delivering parameters [19]. Consequently, the current knowledge of protein-ligand interactions still needs to be improved in order to lower the cost and time of drug discovery and development.

In this work, we motivate and demonstrate the use of rigidity analysis to assess the role that each atom of a ligand has on the stability of the protein-ligand complex. Not only is our approach quick—needing, at most, hours of compute time—compared to wet lab experiments, but it also assesses the role of each atom in the engineered drug, which provides a level of detail that is not easily accessible via most other methods.

## 2. Related Work

**Figure 1:**
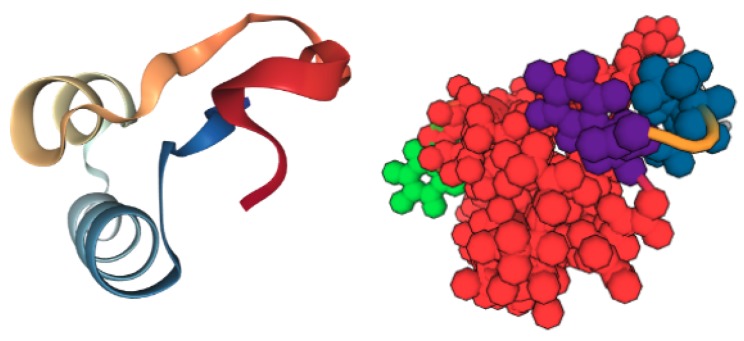
The rigidity analysis of a protein (PDB 1CRN, (**left**)) identifies rigid clusters of atoms (**right**).

Previously, we have relied on rigidity analysis to gain insights about a variety of structural and biochemical properties of biomolecules. Rigidity analysis is a fast combinatorial approach for identifying rigid regions, or clusters, of atoms in a protein [20,21]. Atoms and their chemical interactions are used to construct a mechanical model, from which a graph is generated, on which pebble game algorithms [22] are used to analyze the rigidity of the associated graph and biomolecule (Figure 1). A biomolecule may be composed of many small rigid clusters, one or more larger ones, or a combination of both large and small rigid clusters. In our previous work, we correlated the size and distribution of rigid clusters to a protein’s stability [23] and compared the rigidity properties of the wild type and mutants to infer the effects of amino acid substitutions [24,25].

In this work, we systematically engineer in silico all the possible variants of the ligand in a protein-drug complex, as well as measure the rigidity of all generated complexes as a way to infer how individual atoms in the ligand contribute to the drug’s effect on the protein.

### Rigidity Distance

To quantitatively compare a protein mutant to its wild type, we previously developed the Rigidity Distance (RD) metric [26]. To assess the effects of deleting, in silico, an atom from the ligand, we compare the count and sizes of the different rigid clusters in the wild type of the protein-ligand complex and the protein-ligand mutant, via our $RD_{WT\to Variant}$ metric:

$$RD_{WT\to Variant}:\sum_{i=1}^{LRC}i\times \left[WT_{i}-Var_{i}\right],$$

where WT refers to the non-mutated protein-drug complex, Var refers to a variant of the protein-drug complex in which the ligand is altered in silico, and LRC is the size of the Largest Rigid Cluster (in atoms). Each summation term of the $RD_{WT\to Variant}$ metric calculates the difference in the count of a specific cluster size, *i*, of the wild type and variant, and weights that difference by *i*. The weighting is introduced so that differences among larger clusters of rigid atoms have a greater impact on the RD score, which we found to be the best approach in correlating the RD metric to experimental data [27].

The use of the RD metric permits us to quantitatively assess the extent that each atom in the ligand has on the protein with which it is in complex.

## 3. Methods

**Figure 2:**
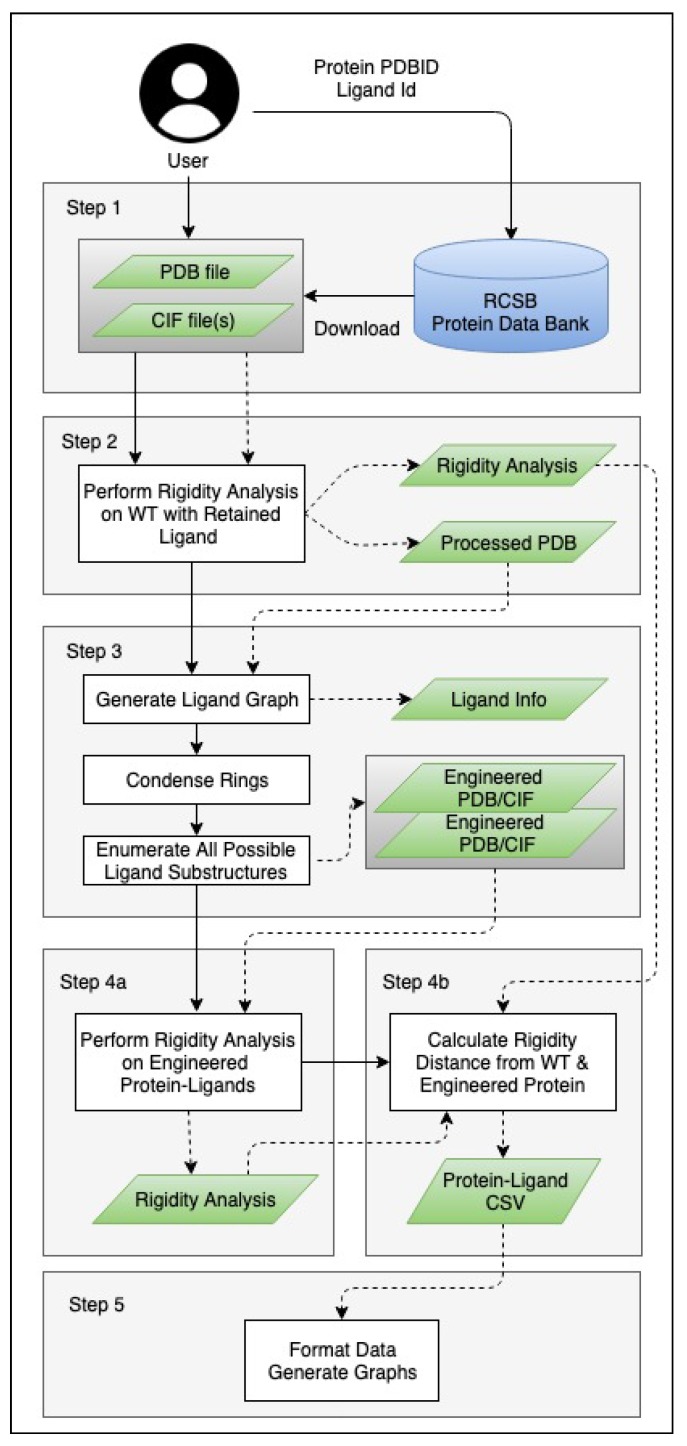
**P**rotein-Ligand complex **E**ngineering **T**hrough **R**igidity **A**nalysis (PETRA) compute pipeline. Dotted lines specify data; solid lines specify control flow.

For this work, we relied on our recently developed computational pipeline, **P**rotein-Ligand complex **E**ngineering **T**hrough **R**igidity **A**nalysis (PETRA). PETRA is a multi-step system that in silico engineers variants of ligand in a protein-drug complex and analyzes each variant using freely available rigidity analysis software [20]. The input to PETRA is either a protein-complex structure file from the RCSB protein data bank [28], or custom user-supplied PDB-formatted and CIF files for a protein-ligand complex. PETRA generates all possible complex variants in which atoms are removed systematically from the ligand. PETRA performs rigidity analysis of the wild type protein-drug complex and all of the variants. The results of the rigidity analyses are analyzed to infer how each atom in the ligand affects the stability of the protein-drug complex (Figure 2).

### 3.1. Generating Ligand Variants

**Figure 3:**
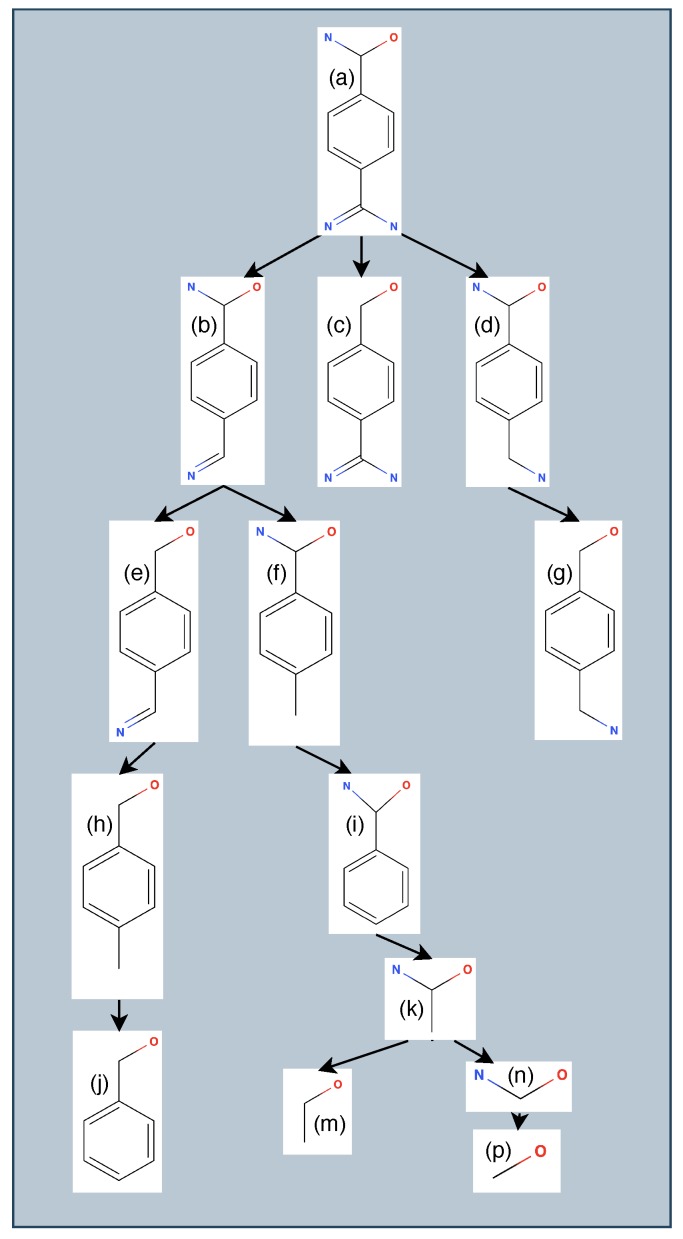
Mock ligand and tree structure paths for generating an example set of 14 ligand variants. The oxygen is the root of the ligand because it engages in a stabilizing interaction with the protein; consequently, the oxygen is a member of each ligand variant. A depth-first traversal of the tree represents successive removals of atoms. Variants (b), (c), (d), for example, are all variants that have one atom less than the ligand that is the root of that tree (a).

To generate ligand variants, PETRA employs a depth-first traversal of a graph representation of the drug compound (Figure 3). Each node in the graph represents a non-hydrogen atom in the ligand. Any ligand atoms that engage in hydrogen bonds or hydrophobic interactions with the protein have their corresponding node marked as a root node for future steps. Our approach for in silico generating variants of a ligand leverages a depth-first search of a tree representation of a drug, in which nodes—representing atoms—are successively pruned away. We have chosen to always retain an atom of the ligand that engages in a stabilizing interaction with the protein because it is these interactions that give rise to the specificity and catalytic properties of a drug. Modifying atoms that engage in those interactions would negate the biochemical reasons why the drug was engineered in the first place. During the pruning process, rings are condensed into single nodes because generating all possible substructures of a ring (for example, removing a single atom from a benzene ring) is not only biologically infeasible but also would result in a cycle in the graph representation of the ligand compound.

From this graph, multiple tree structures are formed, each of which is rooted at any node marked as root in the graph. When a ligand binds multiple ways to the protein via hydrogen bonds or hydrophobic interactions, multiple trees are generated, where each root node represents the atom in the ligand that interacts biochemically with the protein. The tree structure allows for enumeration of all possible sub-trees that contain the root (Figure 3). This is done through a depth-first traversal, with each leaf returning all possible substructures in a set to prevent any duplicate substructures across multiple trees. Note that the ligand modifications process is done without regard of whether the atomic modifications are able to be realized via a synthesis process using existing wet-lab techniques and without regard whether the protein and ligand would still bind. However, each ligand variant retains at least one atom (the root) that engages in a stabilizing interaction with the protein target, and this is done to preserve the atom that is responsible for a biochemical interaction.

### 3.2. Analysis and Visualizations

To better understand the effect of removing an atom from a ligand that is in complex with a protein, we developed two rigidity-based metrics along with visualization of them. The metrics are the average change in rigidity distance (RD) when a single atom is cut from a ligand, and the average rigidity distance (RD) when a pairwise set of atoms is contained in the ligand variant. See Section 2 for an overview of how we and others have used rigidity analysis to explore how point mutations affect the structural stability of a protein.

#### 3.2.1. SingleCut: the Role of Single Atoms

**Figure 4:**
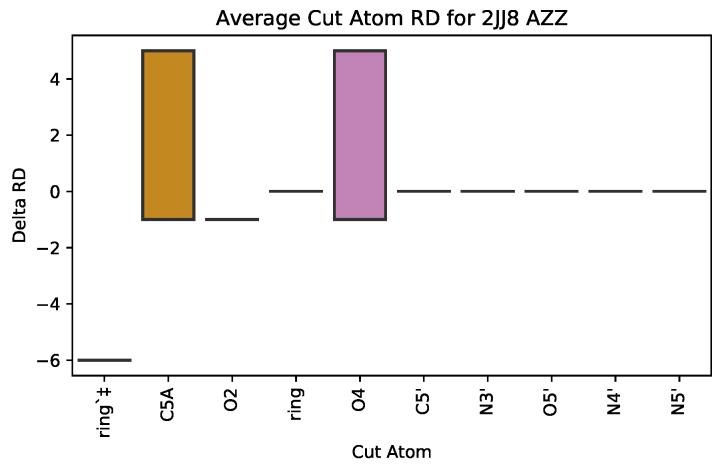
Box plot for $SingelCut_{i}$, Equation (1). The x-axis represents a singular cut atom and y-axis values are the average change in rigidity distance of the protein when the singular atom is cut from a ligand variant. ‡ designates involvement in a hydrophobic interaction.

To better understand how a single atom contributes to the effect that a ligand has on the rigidity of a protein, we calculate the change in rigidity distance for all ligand variants with a specific atom removed from the drug. Note that the removal of atom *x* from a ligand may be the 1st, 2nd, … all the way up to the last atom to be removed, so our metric reasons about a set of ligands. When the change in rigidity distance is high, this indicates that the singular atom in the ligand plays an important role in how the drug affects the stability of the protein. This metric is calculated via the following:

(1) $$SingleCut_{i}=\sum_{i\in \alpha^{\prime }}\frac{\sum_{j=1}^{n}\left\{\begin{array}{cc} \gamma^{\prime } & \mathrm{if}\;i\in j\\ 0 & \mathrm{if}i\notin j \end{array}\right.}{m},$$

where $\alpha^{\prime }$ is the set of all atoms *cut* from a given ligand, *j* is the set of cut atom(s) of the current cut, starting from 1*^st^* cut, going to the $n^{th}$ cut, *m* is the number of times $atom_{i}$ is cut from a ligand variant, and $\gamma^{\prime }$ is the change in rigidity distance (RD) of a protein when an atom is cut, calculated by $RD_{\mathrm{before}\;\mathrm{cut}}-RD_{\mathrm{after}\;\mathrm{cut}}$. We use a box plot (Figure 4) to visualize SingleCut.

#### 3.2.2. PairCut: The Role of Pairs of Atoms

**Figure 5:**
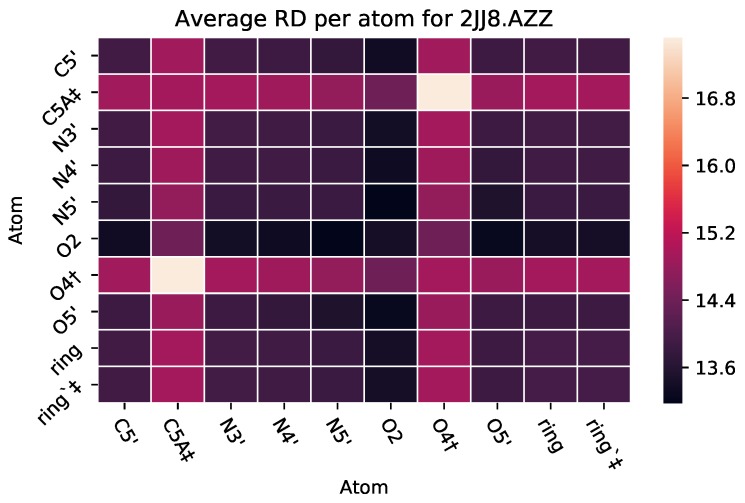
Heat map for PairCut${}_{i,j}$, Equation (2). The x and y axes represent all the atoms in a given ligand, while the colors represent the rigidity distance of a protein when each pairwise set of atoms (i.e., C5${}^{\prime }$ and O2) is contained in a ligand variant. † = Hydrogen Bond ‡ = Hydrophobic Interaction.

To reason how pairs of atoms contribute to the effect that the ligand has on the protein, we take the average rigidity distance of the ligand when a pairwise set of atoms is *contained* within the ligand. We use this metric to acquire a better understanding of the effect of each atom, in combination with another one, and how their combined presence contributes to the ligand’s effect on the rigidity of the protein target. If the pairwise atoms that are contained in the ligand have an average rigidity distance that is relatively low in magnitude compared to the rest of the pair-wise atoms, the atoms contained play an important role in the ligand’s (drug’s) effect on the rigidity of the protein. The PairCut metric lends itself to the use of a heat map (Figure 5). The PairCut metric is provided in the following equation:

(2) $$PairCut_{i,j}=\sum_{i,j\in \beta }\frac{\sum_{k\in \alpha }\left\{\begin{array}{cc} \gamma & \mathrm{if}\;i,j\in k\\ 0 & \mathrm{if}\;i,j\notin k \end{array}\right.}{{\left|\{(i,j,\lambda )|\{i,j,\lambda \}\subseteq \alpha \}\right|}^{\prime }}$$

where β is the set of all atoms in a given ligand, α is the set of all atoms *not cut* at a point in time from a given ligand variant, γ is the rigidity distance of a ligand variant that contains $atom_{i}$ and $atom_{j}$, λ is any combination of atoms contained in a ligand variant (not including $atom_{i}$ or $atom_{j}$), and |{(i,j,λ)|{i,j,λ}⊆α}| is the number of ligand variants containing $atom_{i}$ and $atom_{j}$. Note that rings are treated as singular “atoms” because they are excised as a unit. This equation sums the rigidity distance of each pairwise set of atoms, $atom_{i}$ and $atom_{j}$, and then divides that sum by the number of ligand variants containing $atom_{i}$ and $atom_{j}$ in order to find the average rigidity distance for each pairwise set of atoms.

#### 3.2.3. Protein-Ligand Interactions

**Figure 6:**
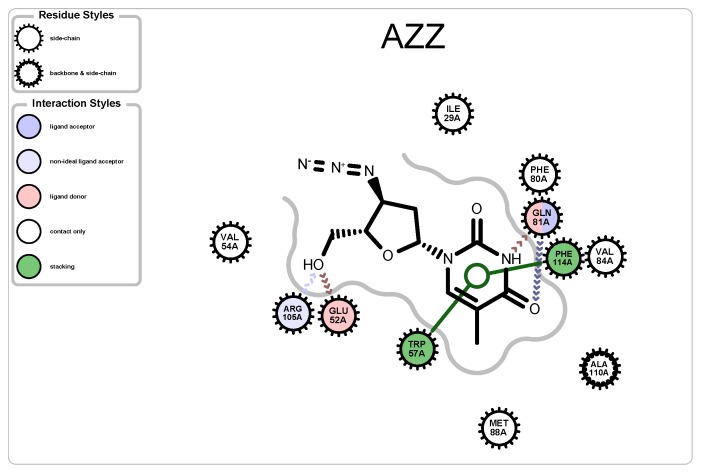
A protein-ligand interaction map generated from the third party software, *OpenEye*, visualizing the interaction between PDB 2JJ8 and ligand AZZ.

To aid in understanding which atoms of the ligand interact with the protein target, we relied on the third-party software *OpenEye* [29] (see example interaction map shown in Figure 6).

## 4. Data Set, Run-Times

Thirty-two protein-ligand complexes were analyzed using PETRA (Table 1). Run-time and data metrics representative of the range of protein-ligand complexes are shown in Table 2. An analysis of PDB 2PJ6, for example, created 7989 ligand variants, generating 5.7 GB of data, and needed 6 hours of compute time on a 6-core 3.7 GHz processor with 16GB in a docker container, enabling 6 ligand variants generated and analyzed concurrently. As can be seen, even large PDB files had reasonable run-times.

## 5. Case Studies and Discussion

We focused on analyzing protein-ligand complexes of well-researched drugs for which details about specific atoms and functional groups are available in the literature. These include Ibuprofen, Zidovudine, Aspirin, and Ciprofloxacin. Here, we present several case studies to demonstrate the utility of PETRA and its use in helping to better understand the roles of individual atoms in the effects that drugs have on protein targets.

### 5.1. Case Study 1: Ciprofloxacin

**Figure 7:**
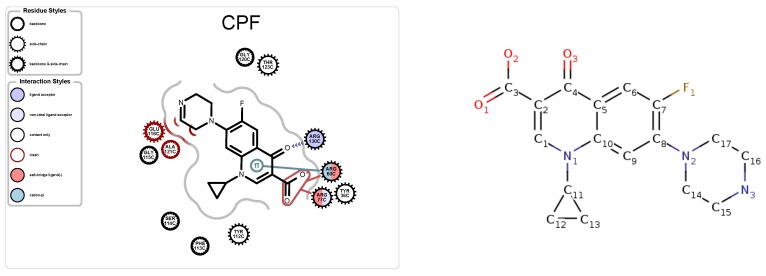
Protein-ligand map (**left**), and schematic (**right**) of Ciprofloxacin, CPF, from PDB 4KRA.

The ligand in PDB file 4KRA, Ciprofloxacin (Figure 7), was first approved as an antibiotic drug in 1987 and is a member of a quinolone group of antibiotics [30]. The structural design of quinolones uses quinine, which features two cyclohexenes. Before Ciprofloxacin was developed, additions to the quinolone group included a fluorine atom and a six-sided ring. Ciprofloxacin retains the design of the previous attempts but added a cyclopropyl group.

**Figure 8:**
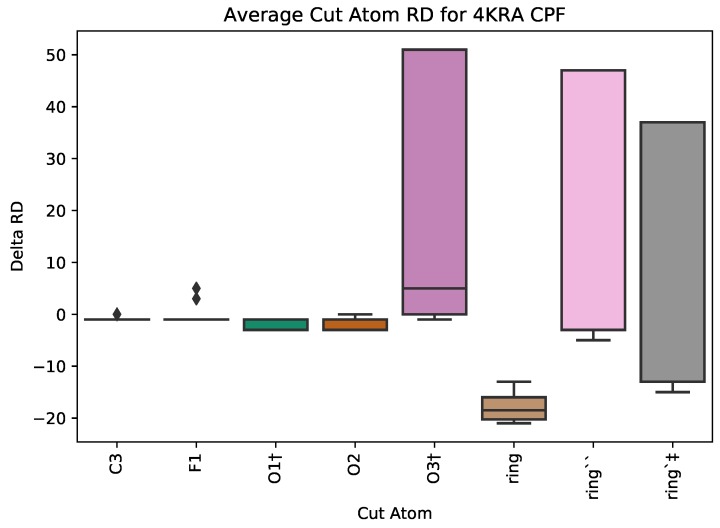
SingleCut metric for PDB 4KRA in complex with CPF. ring = C2,C1,N1,C10,C9,C8,C7,C6,C5,C4, ring${}^{\prime }$ = N2,C17,C16,N3,C15,C14, ring${}^{\prime \prime }$ = C11,C12,C13. † and ‡ designate atoms engaging in hydrogen bonds and hydrophobic interactions, respectively.

Using PETRA, we assessed the effects that each addition to the basic foundation of the design quinolone group has on the rigidity on the protein-ligand complex (Figure 8). When atom O3 is cut from Ciprofloxacin, it induces the largest change, RD=51, indicating that atom’s significant role in the effect that the drug has on the protein target. Indeed, the 03 atom is needed for gyrase binding and bacterial transport [30]. The two other oxygen atoms O1 and O2 (which are bonded to C3) are involved in gyrase binding and bacterial transport, and they have a small positive effect on the median rigidity distance. The important addition that distinguishes Ciprofloxacin is that the cyclopropyl group can have the second largest change of 47 in rigidity distance. Biochemically, this cyclopropyl was designed to increase the potency of the drug. Thus, the use of PETRA, and it revealing the importance of the O3 atom, appears to be corroborated by biochemical experiments performed in wet-lab settings.

### 5.2. Case Study 2: Human Serum Albumin

**Figure 9:**
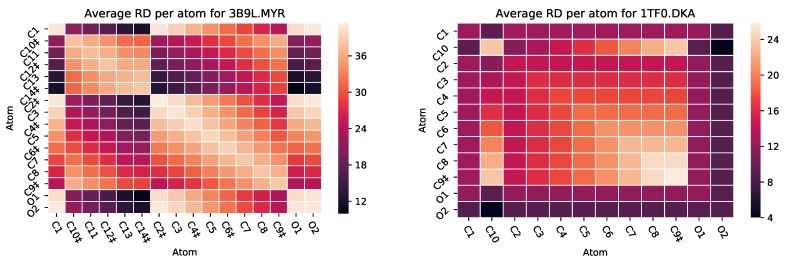
$PairCut_{i,j}$ heatmaps for Myristic Acid in complex with Human serum albumin (HSA) ((**left**), PDB 3B9L), and in complex with Decanoic Acid ((**right**), PDB 1TF0). ‡ designates an atom engaging in hydrophobic interactions.

Human serum albumin (HSA) is a highly abundant lipid binding protein found in blood. HSA has many functions including transportation of medicine through the body [31]. Myristic Acid (MYR) and Decanoic Acid (DKA) are two fatty acid ligands that are known to bind to HSA [32]. For an analysis of two ligands and their effect on HSA, we analyzed PDB files 3B9L and 1TF0. In wet-lab studies [32], Decanoic Acid does not induce a conformational changes in HSA, perhaps due to its short length. MYR, however, has more molecules that bind to HSA and, indeed, it induces a conformational change. In the heat maps generated from the PairCut analysis using PETRA (Figure 9), MYR has a larger overall change in RD, meaning the wildtype HSA has more of a change in its rigid cluster when atoms are removed from the ligand. Future studies will assess whether a high change in RD correlates whether a ligand will or will not produce a conformational change in the protein.

**Figure 10:**
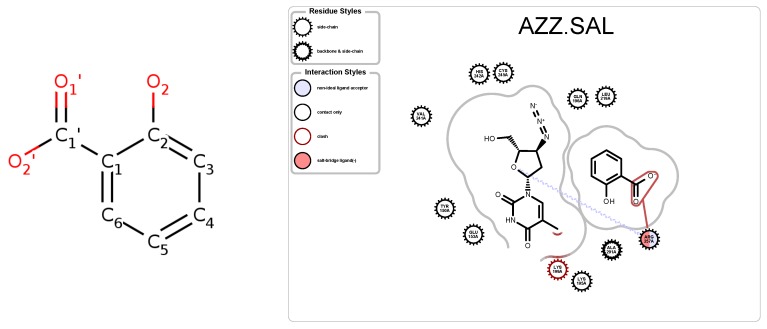
Salicyclic Acid (**left**), and schematic (**right**) of AZT and SAL in complex with HSA, PDB 3B9M.

**Figure 11:**
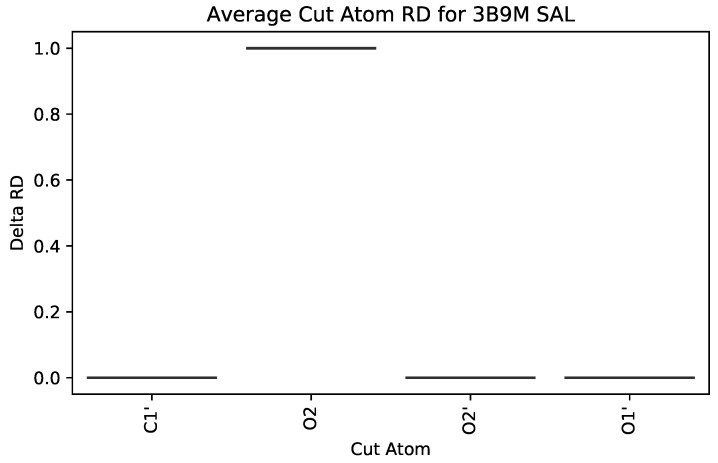
Box plot of SingleCut metrics for atoms in Salycylic Acid, in complex with HSA, PDB 3B9M. The flat quartile boxes designate no variance when cut.

Salicylic acid (SAL, commonly known as aspirin, Figure 10) also binds to HSA. The hydroxyl group (O${}_{2}$) participates in a hydrogen bond with one of the pocket it binds to [32]. Our visualizations highlight the effect of O${}_{2}$ on HSA as it is the only atom that has a noticeable effect as determined via the SingleCut metric. In Figure 11, the lines represent the box plots with minimal quartiles; nonetheless, only when O${}_{2}$ is cut, does the RD change.

**Figure 12:**
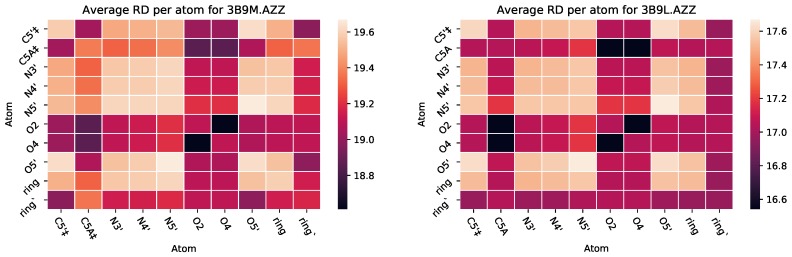
Heat maps for PairCut metrics for HSA in complex with AZT and Myristic Acid (MYR) and SAL ((**left**), PDB 3B9M), and in complex with just AZT and MYR ((**right**), PDB 3B9L). ‡ designates hydrophobic interactions.

It has been shown that Zidovudine (AZT or AZZ) binds differently to HSA depending on whether it is in complex with just MYR or MYR and SAL [32]. SAL out-competes AZT in its ability to bind, meaning that AZT has more molecules that bind with HSA when only with MYR. Figure 12 represents the PairCut metrics for two PDB files, containing HSA in complex with different ligands. PDB file 3B9L is for HSA in complex with AZT and MYR, while 3B9M is HSA in complex with AZT and MYR and SAL. The average RD overall shown is 2 units lower for the HSA-AZT-MYR complex than the HSA-AZT-MYR-SAL complex. Although this difference in RD metrics is small, it is in agreement with wet-lab experiments reported in the literature.

### 5.3. Case Study 3: Ibuprofen

**Figure 13:**
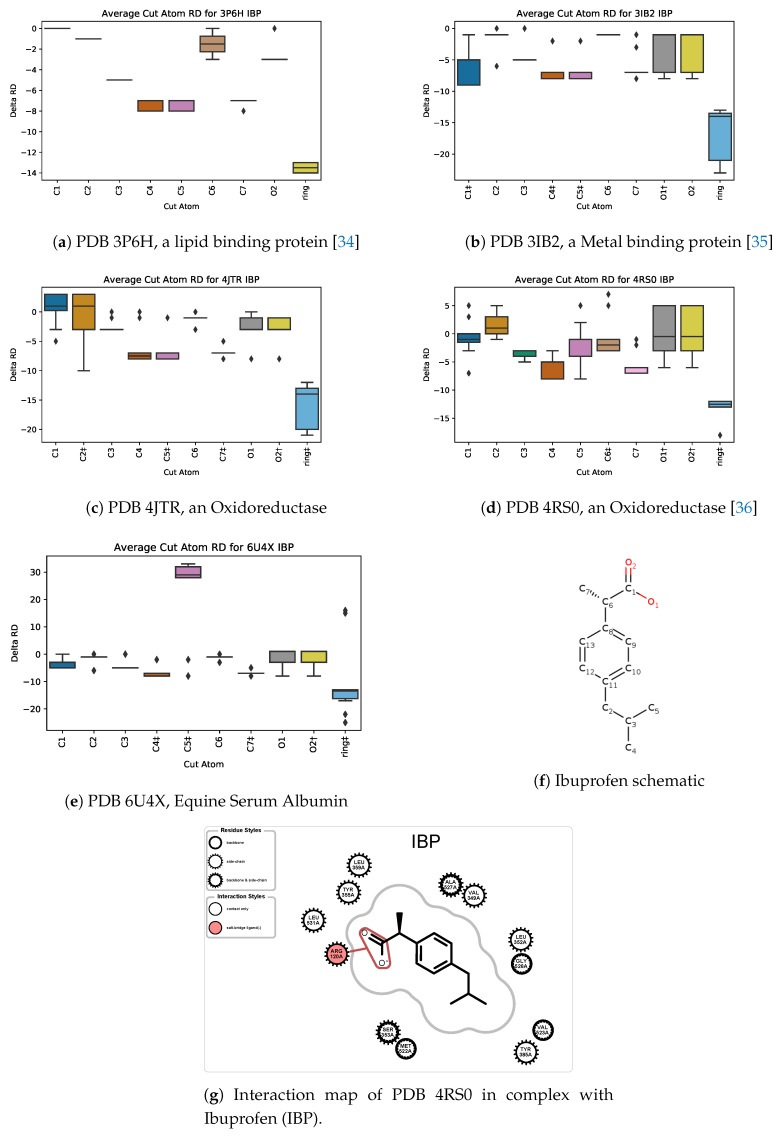
Ibuprofen (IBP) schematic and sample interaction map, and box plots for *SingleCut* metrics for 5 protein-IBP complexes. † and ‡ identify atoms involved in hydrogen bond and hydrophobic interactions, respectively.

Ibuprofen (CIF ID: IBP) is a chiral nonsteroidal anti-inflammatory drug (NSAID) [33] and is a common household item. We used PETRA to analyze five different protein-ligand complexes with ibuprofen as the ligand (Figure 13).

The aromatic ring had the greatest average rigidity distance when it was removed from the ligand in all 5 of the complexes run except for PDB ID 6U4X (Figure 13e), which is equine serum albumin. This could be interpreted that the aromatic ring has the greatest impact on the rigidity of the protein. Indeed, Gonzalez and Fisher [34] have found that the aromatic ring greatly affects the positioning of Ibuprofen in a human adipocyte lipid-binding protein named FABP4 (PDB 3P6H, Figure 13a). Thus, the aromatic ring can increase the ligand’s ability to bind to the protein in a hydrophobic environment. The second protein PDB 3IB2 (Figure 13b) demonstrates this, in which also ibuprofen binds in a cleft with six hydrophobic residues [35]. The third protein PDB 4JTR (Figure 13c), which is a COX-2 mutant, also binds ibuprofen in a hydrophobic environment [36].

For the fourth protein (PDB 4RS0, an AKR1C2 complex shown in Figure 13d), the manuscript is not yet available (to be published by Yosaatmadja et al.). Nonetheless, the SingleCut metric plot identifies the aromatic rings as contributing significantly to the ligand’s effect, in corroboration with our other analyses, revealing that the aromatic ring has a large effect on the ligand’s binding ability.

For the case of PDB 6U4X, atom C5, which is among one of the first atoms removed by PETRA due to its location on the periphery of the drug, has the largest rigidity distance change when it is removed. This could be a sign that C5 plays an important role in how ibuprofen binds to the equine serum albumin. The manuscript accompanying 6U4X similarly has not yet been published (Czub et al.); therefore, the significance of C5 is not yet corroborated by wet-lab work. Nonetheless, the SingleCut metric box plot for 6U4X (Figure 13e) identifies C5 is the only atom with a positive average RD when retained in the ligand.

### 5.4. Case Study 4: Ibuprofen Variants

**Figure 14:**
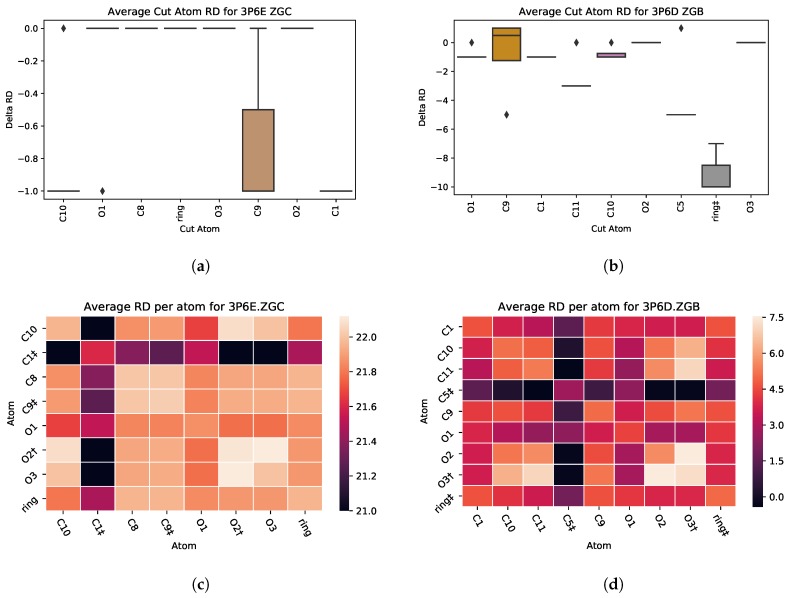
PDB 3P6D-ZGB contains a variant of Ibuprofin with its aromatic ring containing a methyl group (*SingleCut* in (**a**), *PairCut* in (**c**)), while PDB 3P6E-ZGC does not include the methyl group (*SingleCut* in (**b**), *PairCut* in (**d**)).

A case in which PETRA is not able to predict the importance of an atom’s contribution to the ligand’s binding affinity is with two complexes which include variants of ibuprofen. PDB files 3P6D and 3P6E contain ligands that are based of the structure of ibuprofen. The only difference between the two is the attaching of a methyl group to the aromatic ring [34]. This methyl group causes a 100-fold increase in the binding affinity of the ligand. The methyl group is represented as C1 in Figure 14b, which shows minimal effect on the RD metric when removed from the ligand. Furthermore, the PairCut heat maps in Figure 14b indicate that the average RD is higher in 3P6E, which is without the methyl group, which is opposite of what would be expected considering the 100-fold increase.

## 6. Conclusions and Future Work

Pharmaceutic drug engineering efforts are expensive and time-consuming. Although a variety of software tools are available for exploring and aiding the drug design process, none employ the use of rigidity analysis to explore how each atom of a ligand contributes to the drug’s effect on the structural stability of a protein target. We developed a compute pipeline, PETRA, that for a PDB file of a protein-ligand complex, generates in silico all possible ligand variants. Each variant is assessed to help infer the role of individual atoms of the ligand. We have shown via case studies and visualizations that PETRA can provide insights about the roles of the atoms in the ligand, and this is corroborated by wet-lab studies reported in the literature.

In ongoing work, we are continuing to assess the predictions output by PETRA against the efficacy rates of drugs as determined via clinical trials and in wet-lab assay experiments. The next several steps are envisioned or are ongoing, including the use of machine learning methods to develop accurate predictions of the effects of atoms in a ligand. PETRA is being prepared for public release via an open source Docker container that manages dependencies and custom wrapper software for file input and output options. The source code for PETRA is available upon request.

## Figures and Tables

**Table 1:** Twenty-two of the 32 protein-ligand complexes analyzed using PETRA.

PDB ID	Ligand	Protein Res.	HBs and HIs	Num Rings	Largest Change in RD	Largest Rigid Cluster
1H9Z	RWF	582	5	2	−16	1334
1HA2	SWF	582	7	2	21	1041
1TF0	DKA	609	1	0	−8	3703
2BXD	RWF	1156	9	2	−23	1700
2I2Z	SAL	581	1	1	1	1059
2I2Z	MYR	581	8	0	−8	1059
2JJ8	AZZ	736	6	2	−6	2570
3B9L	MYR	582	7	0	−7	1218
3B9L	AZZ	582	1	2	−9	1218
3B9M	SAL	582	1	1	1	1092
3B9M	MYR	582	6	0	−7	1118
3B9M	AZZ	582	2	2	−9	1092
3BCR	AZZ	812	2	2	3	6804
3IB2	IBP	341	4	1	−23	2651
3P6D	ZGB	138	4	1	−10	1422
3P6E	ZGC	139	3	1	−1	1342
3P6G	IZP	139	3	1	−3	1405
3P6H	IBP	139	1	1	−14	1475
4JTR	IBP	633	5	1	−21	5381
4KRA	CPF	1018	5	3	51	7271
4RS0	IBP	557	4	1	−18	5524
6U4X	IBP	580	9	1	33	3050

**Table 2:** Run-time and data metrics for a representative sample of the protein-ligand complexes we analyzed. These were run on a 6-core 3.7 GHz processor with 16 GB in a docker container, with 6 ligand variants generated then analyzed concurrently.

PDB ID	Num. of Residues	Ligand	Ligand Size	Num. of Lig Variants	Run Time (min)	Data Size
2PJ6	306	059	32	7989	641	5.7 GB
2RKN	77	LP3	30	163	1.0	41 MB
3CYW	198	017	38	3293	104	2.2 GB
3IWL	68	TCE	16	367	1.8	74 MB
3JVY	198	017	38	3293	109	2.1 GB
4KRA	1023	CPF	24	72	53	147 MB
